# TFEB Promotes Prostate Cancer Progression *via* Regulating ABCA2-Dependent Lysosomal Biogenesis

**DOI:** 10.3389/fonc.2021.632524

**Published:** 2021-03-01

**Authors:** Xuejin Zhu, Yangjia Zhuo, Shulin Wu, Yanfei Chen, Jianheng Ye, Yulin Deng, Yuanfa Feng, Ren Liu, Shanghua Cai, Zhihao Zou, Bin Wang, Chin-Lee Wu, Guohua Zeng, Weide Zhong

**Affiliations:** ^1^Department of Urology and Guangdong Key Laboratory of Urology, The First Affiliated Hospital of Guangzhou Medical University, Guangzhou, China; ^2^Department of Urology, Guangdong Key Laboratory of Clinical Molecular Medicine and Diagnostics, Guangzhou First People’s Hospital, Guangzhou Medical University, Guangzhou, China; ^3^Department of Urology, Guangdong Key Laboratory of Clinical Molecular Medicine and Diagnostics, Guangzhou First People’s Hospital, School of Medicine, South China University of Technology, Guangzhou, China; ^4^Department of Urology, Massachusetts General Hospital, Harvard Medical School, Boston, MA, United States; ^5^Department of Pathology, Massachusetts General Hospital, Harvard Medical School, Boston, MA, United States; ^6^Department of Urology, Affiliated Cancer Hospital & Institute of Guangzhou Medical University, Guangzhou Medical University, Guangzhou, China

**Keywords:** TFEB, ABCA2, prostate cancer, tumor microenvironment, lysosomal biogenesis, biochemical recurrence, metastasis

## Abstract

Transcription factor EB (TFEB), a member of the MiT family, is dysregulated in different cancers and exerts specific biological functions within the tumor microenvironment. Downregulation of TFEB induces macrophage polarization in the TME and promotes tumor progression. However, the biological role and clinical significance of TFEB in prostate cancer (PCa) remain unknown. This study aimed to identify the role of TFEB in PCa and its potential clinical value. We explored TFEB expression in PCa using public databases and verified its prognostic value using immunohistochemistry in PCa tissue samples. The results revealed that TFEB expression was up-regulated in PCa tissues and was associated with cancer metastasis. Next, overexpression of TFEB promoted PCa cell malignant behavior in *in vivo* and *in vitro* experiments. RNA-sequencing and bioinformatics analysis showed high expression of TFEB promoted lysosomal biogenesis and knockdown of TFEB expression decreased the number of lysosomes. Furthermore, the ATP-binding cassette transporter A2 (ABCA2) was identified as a target gene of TFEB, which was verified using the cleavage under targets and release using nuclease (CUT&RUN) assay and qRT-PCR. Silencing of ABCA2 reduced lysosomal biogenesis and decreased matrix metalloproteinases expression, which reduced PCa cell invasion and migration in the tumor microenvironment. Our study suggests that TFEB promotes PCa progression by regulating ABCA2 through lysosomal biogenesis and may serve as a prognostic factor or as a potential therapeutic target of PCa.

## Introduction

Prostate cancer (PCa) remains a leading cause of cancer incidence and mortality in the United States, with 191,930 new cases and 33,300 deaths reported annually (1). In the past decade, the frequency and mortality of PCa in China has exhibited an increasing course (2). The principal modality in treating localized PCa is radical prostatectomy (3). Although most PCa patients can initially receive RP, many will progress to biochemical recurrence and/or metastasis (4, 5). Presently, the preoperative serum prostate-specific antigen (PSA), the Gleason score (GS), TNM stage, and surgical margin status are the predominant methods for predicting PCa prognosis. However, these indexes, with limitations in the differentiation of the biological heterogeneity of tumors, cannot precisely estimate the risk of aggressive PCa. Therefore, identifying novel sensitive and specific biomarkers to monitor the prognosis of PCa is urgently required.

Transcription factor EB (TFEB, also known as BHLHE35 or ALPHATFEB), a member of the microphthalmia family (MiTF/TFE family), is a master transcriptional regulator of lysosomal biogenesis and autophagy and regulates the expression of various lysosome and autophagy genes (6). In the breast tumor microenvironment (TME), macrophage-specific TFEB knockout promoted breast tumor growth by inducing macrophage M2 polarization through autophagy/lysosome-mediated pathways (7). Dysregulated TFEB has been implicated in many human diseases, including several types of solid tumors, neurodegenerative diseases, and lysosomal diseases (8). Furthermore, TFEB is highly expressed in glioblastoma (9), non-small lung cancer (10), pancreatic ductal adenocarcinoma (11), and breast carcinoma (12). Enhanced TFEB expression correlates with aggressive clinical features and can be an unfavorable independent prognostic factor in breast cancer. In PCa, a previous study found that the androgen receptor can increase TFEB expression by binding to the promoter region (13). However, the expression of TFEB in the human prostate TME and its biologic role and clinical significance remain unknown.

The lysosome is a crucial catabolic membrane-bound organelle. There are various hydrolases in the lysosomal microenvironment, those hydrolases can decompose complex macromolecules, such as large protein complexes, nucleotides, lipids, glycolipids, and glycoprotein, recycle the endocytic receptor, and participate in energy metabolism (14, 15). Normal lysosomal biogenesis levels are essential for cells to sustain a healthy intracellular environment, whereas tumors with increased lysosomal biogenesis have been correlated with highly invasive and metastatic behavior and, ultimately, poor prognosis (12). There are many kinds of cysteine cathepsins active in the lysosome, which can be exocytosed to the TME and degrade the extracellular matrix to promote cancer cell invasion and migration (16). Furthermore, cancer cells lysosomes can degrade chemotherapeutic drugs through internal acid hydrolase to obtain chemotherapy resistance as treatment time increases (17).

In the present study, we aimed to explore the expression and effects of TFEB in PCa. Moreover, the specific biological role of TFEB in PCa were explored using a series of *in vivo* and *in vitro* experiments.

## Materials and Methods

### Patients and Tissue Samples

For immunohistochemistry (IHC) analysis, 205 samples were used for prostate tissue micro-array (TMA) analysis in this study. Detailed information is provided in the **Supplementary Methods**. The data relative to TFEB mRNA expression derived from The Cancer Genome Atlas (18) and was analyzed using UALCAN analysis tools (http://ualcan.path.uab.edu/) (19). To further evaluate the clinical relevance and survival analysis of TFEB downregulated genes, publicly available datasets of prostate tissue mRNA expression were extracted from Taylor dataset (20) s and analyzed by the cBioPortal tool (http://www.cbioportal.org/) (21, 22).

### Immunohistochemical Analysis

Protein expression levels of TFEB and ABCA2 in PCa and xenograft tumor samples were detected by immunohistochemistry. A standard immunoperoxidase staining procedure was used to perform IHC analysis. Primary antibodies against TFEB (A303-673A; BETHYL, TX, USA) or ABCA2(A16735; ABclonal, Wuhan, China) were used at a concentration of 1:200 and 1:50, respectively. The intensity of immunostaining was graded as 0 (negative), 1 (weak), 2 (moderate), and 3 (strong). Two independent authors (XZ and YZ) scored the stained TMA slide in a blinded fashion without any information regarding the patients’ clinicopathological data and clinical outcomes. For cases which the primary reviewers could not reach consensus, a third investigator (CLW) was consulted to reach group consensus. Cases were defined as a high expression if immunostaining intensity was equal to or greater than moderate in > 20% of the cancer cell population.

### Cell Line Culture and Transfection

Four PCa cell lines (22RV1, LNCaP, PC3, and DU145) were purchased from the American Type Culture Collection (USA). The PCa cell lines were grown in RPMI 1640 medium (HyClone, USA) or DMEM high glucose medium (HyClone, USA) supplemented with 10% fetal bovine serum (FBS) (Gibco, USA) and 1% penicillin-streptomycin at 37°C and 5% CO2. TFEB short hairpin RNAs (shTFEB), the scramble shRNA control, the TFEB overexpression plasmid, and the control plasmid were purchased from HYY Med Company (Guangdong, China). The target sequences of the shRNAs were as follows: sh-TFEB#1: 5′-TGGCAACAGTGCTCCCAATAG-3′, sh-TFEB#2: 5′-CGATGTCCTTGGCTACATCAA-3′ and sh-TFEB#3: 5′-GGAGACGAAGGTTCAACAT-3′. TFEB knockdown lentivirus was created by transfection of 293T cells (China Center for Type Culture Collection, Wuhan, China) with packaging plasmid and TFEB knockdown plasmid. TFEB overexpression lentivirus was created by transfection of 293T cells with packaging plasmid and TFEB overexpression plasmid. Then, the different 293T cells were incubated for 48–72 h at 37°C and 5% CO2. Collected lentiviral supernatants were filtered through a 0.45-μm filter (Millipore, America), and then the lentivirus supernatants were added to different PCa cells in the presence of polybrene (1:1,000), respectively. 22RV1 and LNCaP cell lines were transfected with the shTFEB lentivirus, cells transfected with scramble vector lentivirus were used as controls; DU145 and PC3 cells were transfected with the TFEB overexpression lentivirus, also DU145 and PC3 cells transfected with vector lentivirus were used as controls. Stable cell lines were generated using puromycin selection (2 μg/ml) for 48 h after transfection, levels of TFEB were measured by western blotting. The ABCA2‐siRNA and negative control siRNA were synthesized and modified by Guangzhou RiboBio (RiboBio, China).

### Western Blot Analysis

Cells or tissues were lysed in RIPA, PMSF, and SDS buffer. Ten percent of SDS-PAGE was used to resolve an equal amount of protein, and protein was transferred to a 0.45 um PVDF membrane (Millipore, #ISEQ00010) and then blocked for one and half hours at room temperature (RT) with 5% phosphate-buffered saline tween-20 (PBS-T) milk. Membranes were incubated overnight with primary antibodies. TFEB (A303-673A) was obtained from Bethyl Laboratories (TX, USA). ABCA2 (A16735) was obtained from ABclonal (Wuhan, China). Antibodies are specific for LAMP1 (21997-1-AP), GAPDH (60004-1-Ig), β-actin (66009-1-Ig), and TBP (66166-1-Ig) were purchased from Proteintech Group, Inc. (Chicago, IL, USA). Secondary antibodies, HRP-conjugated goat anti-rabbit IgG (H+L) antibodies (SA00001-2), and goat anti-mouse IgG (H+L) antibodies (SA00001-2) were purchased from Proteintech Group, Inc. (Chicago, IL, USA). ECL Western blotting reagent (Thermo Pierce) was used to visualize the proteins. The Nuclear/Cytosol Fractionation Kit (BB-36021, BestBio Science, Shanghai, China) was used to extract the cytoplasmic and nuclear proteins according to the manufacturer’s procedure. Western blot data quantification was analyzed by ImageJ (National Institutes of Health, MD, USA).

### RNA Extraction, Semiquantitative RT-PCR, and qPCR

RNA extraction, semiquantitative RT-PCR, and qPCR were performed as described previously. Briefly, total RNA from prostate tissue samples and prostate cancer cells was purified using TRIzol (15596-026, Invitrogen, CA, USA) and prepared with HiScript III RT SuperMix for qPCR (+gDNA wiper) (R323-01, Vazyme, Nanjing, China). Complementary DNA (cDNA) was synthesized using the ChamQ Universal SYBR qPCR Master Mix (Q711-03, Vazyme, Nanjing, China). Reactions were run on a CFX96 Touch™ Real-Time PCR Detection System (Bio-Rad, CA, USA). The relative mRNA expression levels of target genes were calculated by the 2^−ΔCT^ method. The corresponding genes in control cells were used to define as a baseline. The PCR products were analyzed by electrophoresis on 1.5% agarose gel. β-actin was used as an internal control of RNA integrity, and the assay was always performed in triplicate. Detailed information about the primer sequence is provided in **Supplementary Methods**.

### Cell Viability, Migration, and Invasion Assays

Cell counting kit 8 (CCK8), transwell, and wound-healing assay were performed to estimate PCa cell viability, invasion, and migration ability. Details regarding these assays are based on our previous studies (23–25).

#### CCK8 Assay

The CCK8 assay kit (MA0218, Meilunbio, Dalian, China) was used to test cells’ proliferation. Approximately 2,500 cells were planted into 96-well plates and cultured for 4, 24, 48, 72, and 96 h with complete medium. Then cells were then incubated with 90 μl medium and 10 μl CCK-8 for 2 h at 37°C. The OD value of 96-well plates was measured by a spectrophotometer (iMark™, Bio-rad, CA, USA) at 450 nm wavelength. The data were presented as the results of three independent experiments.

#### Transwell Assay

In the chamber’s upper compartment, 50,000 cells were seeded suspended in 100 μl of serum-free medium and 500 μl of growth medium containing 10% FBS was added to the lower chamber. After 24 h of incubation, the cells on the upper side of the membrane were removed with a cotton swab. The membrane was fixed with 3.7% paraformaldehyde and then stained with 0.1% crystal violet at room temperature for 1 h. Photos were taken using a microscope camera of the invading cells and the number of cells in four random fields of view were counted.

#### Wound-Healing Assay

The PCa TFEB-knockdown cell lines and TFEB-overexpressed cell lines were seeded in six-well plates separately and grown to nearly 80% confluence for the migration assay. Before using 200 μl sterile tips to make scratches on each well, all wells were cultured with standard medium without FBS. A microscope camera was used for all wells to take photos of healing of scratches every 24 h.

#### Colony Formation Assay

TFEB-overexpressing vector, TFEB-knockdown (shTFEB), or scramble shRNA control (shNC) cells were seeded in six-well plates, 500 cells in each well. PCa cells were treated with complete RPMI1640 or DMEM medium for 14 days. The medium was replaced every 48 h. Next, 0.1% crystal violet was used to fix and stain the cell lines at room temperature for 1 h.

### RNA-Seq and Bioinformatic Analysis

RNA-sequencing of DU145-TFEB and DU145-vector transfected cells was performed by the Novogene Corporation (Beijing, China). Collection, preparation, and library preparation of mRNA samples was performed according to the manufacturer’s protocol. Briefly, total mRNA was obtained using the RNeasy Mini Kit (Qiagen). RNA concentration was measured using the Qubit^®^ RNA Assay Kit in Qubit^®^2.0 Fluorometer (Life Technologies, CA, USA). Following the manufacturer’s recommendations, the NEBNext^®^ UltraTM RNA Library Prep Kit for Illumina^®^ (NEB, USA) was used to generate sequence libraries. Differential expression analysis of DU145-TFEB and DU145-vector cells was performed using the DESeq2 R package (1.16.1); the cutoff values were |log2(fold change)|>1.5 and q<0.05. Metascape (https://metascape.org/gp/index.html) was used to perform KEGG functional enrichment analysis and GESA analysis (26). The GENEMANIA analysis tool (http://genemania.org/) was used to analyze the gene expression pattern (27).

### Lysosomal Staining and Flow Cytometry

Lyso-Tracker was used to estimate the number of lysosomes and lysosomal function. Lyso-Tracker Red DND-99 (C1046) was purchased from Beyotime Biotechnology (Shanghai, China). The fluorescence intensity was observed under a ZESIS 880 confocal microscope (Zeiss, Oberkochen, Germany), and representative cells were selected and photographed. For live-cell Lyso-Tracker flow cytometry analysis, cells were grown to about 80% confluence and treated with Lyso-Tracker Red DND-99 for 1 h. Following trypsinization with 0.05% trypsin EDTA (PYG0014, Boster, Biological Technology, Ltd. Wuhan, China) and resuspended in PBS for FACSCalibur Flow Cytometry (BD Biosciences, USA). Flow Cytometry analysis was performed using FlowJo software (OR, USA).

### Immunofluorescence

For immunofluorescence studies, 5×10^4^ cells/well were seeded on the confocal dish (BS-20-GJM; Biosharp, Hefei, China). After 48 h, cells were treated and fixed with 3.7% paraformaldehyde, washed with PBS, and permeabilized with Triton X-100 for a half-hour, followed by incubation blocking solution (1% BSA) for 1 h. Cells were incubated with the following antibodies diluted in PBS with 1% BSA overnight at 4°C: anti-TFEB (dilution 1:500) and anti-ABCA2 (dilution 1:100). The cells were washed with PBS and incubated with rhodamine (TRITC) goat anti-rabbit IgG (H+L) (AS040, ABclonal, Wuhan, China) for 1 h at room temperature. Cell nuclei were stained with Hoechst 33342 (P0133, Beyotime Biotechnology, Shanghai, China). Labeled cells were examined under the ZESIS LSM880 microscope with a ×100/1.4 objective lens. Confocal microscopy images were acquired and processed with LSM880 system confocal microscope software.

### Transmission Electron Microscopy

Using EDTA for digestion, 22RV1 and DU145 cancer cells lines were collected in 1.5 ml tubes, washed three times with cold PBS buffer and centrifuged at 4°C. After removing the supernatant, the cells were fixed with 2.5% glutaraldehyde in 0.1 M phosphate buffer. Next, cells were fixed and dehydrated in a classified ethanol series and embedded in resin. Leica UC-7 was used to collect ultrathin sections. PCa cells were observed under the transmission electron microscope.

### PCa Cell Lines Xenograft Model

Fourteen nude mice (BALB/c-nu, males, 4–8 weeks old) were purchased from the Sun Yat-sen University Experimental Animal Center (Guangzhou, China). All animals were maintained under specific pathogen-free (SPF) conditions, and all the laboratory animal studies were submitted to and approved by the Research Ethics Committee of Guangzhou Medical University (Guangzhou, China). TFEB-knockdown cells and shNC cells (3×10^6^) were injected subcutaneously into the anesthetized nude mice’s dorsal region. In addition, cells overexpressing TFEB and vector cells (2×10^6^) were injected subcutaneously into the anesthetized nude mice’s dorsal region. Tumor volume (cm^3^) was measured every 4 days once the tumors were measurable, and tumor weight (mg) was measured at the end of the experiment. Nude mice will be euthanized within the specified time, although nude mice in poor condition were euthanized early.

### Transcription Factor Binding Site Analysis and Prediction

The TFEB motif sequence was obtained from the JASPAR database (jasper.genereg.net) (28). For searching transcription factor potential binding sites of target genes promoters, the EPDnew database (https://epd.epfl.ch) (29) was used to get putative transcription factor binding motifs on ABCA2 genes promoters. Promoter regions were defined as the genomic interval from −1900 to +100 bp relative to the putative transcription start sites (TSS).

### Cleavage Under Targets and Release Using Nuclease Assay

CUT&RUN assay (Cell Signaling Technology, MA, USA) of TFEB was performed according to published protocol with modifications (30). Briefly, 8×10^5^ DU145-TFEB cells were collected in a new 1.5 ml tube. After washing with 1x wash buffer (including spermidine and protease inhibitor cocktail) and centrifuging at 700 g for 5 min at room temperature, DU145-TFEB cells were then resuspended with 400 μl buffer. Then 100 µl of above cells were transferred to a new 1.5 ml tube and stored at 4°C, which were taken as input sample for further verification. Another 300 μl DU145-TFEB cells were divided equally and transferred into three new tubes respectively, these three new tubes were marked as the positive control group, negative control group, and TFEB group. Ten microliters of activated concanavalin A-coated magnetic beads suspension was added into these groups for capture cells. These groups were incubated with antibodies targeting Tri-methyl-histone H3, IgG control, and TFEB at 4°C overnight. Tri-methyl-histone H3 and IgG antibody were served as the positive and negative control, respectively. Protein A-MNase enzyme was added into immunoprecipitated samples and incubated for 60 min at 4°C. Then, 2 mM CaCl_2_ was added into immunoprecipitated samples to activate protein A-MNase on ice for half hour. One hundred milliliters of stop buffer was added into these samples to stop the reaction at 15 min at 37°C, then these samples were centrifugated at 4°C and the supernatant containing the CUT&RUN fragments were collected. DNA of CUT&RUN fragments was purified by DNA Purification Kit (Cell signaling technology Inc, MA, USA). For qRT-PCR verification, positive control group, negative control, input DNA group, and experimental group were used to determine downstream genes binding sites capacity and efficiency of amplification.

### Statistical Analyses

All statistical analyses were performed with SPSS v.26.0 (SPSS Inc, Chicago, USA). Continuous variables and data were expressed as means ± SD. Associations between gene expression and clinicopathological characteristics were evaluated by two independent samples t-test and the one-way ANOVA test. Survival rates were calculated using the Kaplan-Meier method, and the log-rank test was used for comparisons. Univariate analysis was performed to evaluate the association between gene expression and oncological outcomes, variables, which showed statistical significance were further fixed into a multivariate Cox proportional hazards model. The relative risks for death outcomes were expressed as adjusted hazard ratios (HRs) and corresponding 95% confidence intervals (CIs). All statistical tests were two-sided, with p < 0.05 considered statistically significant.

## Results

### TFEB Was Upregulated in Patients With PCa and Affected PCa Progression

**Figure 1:**
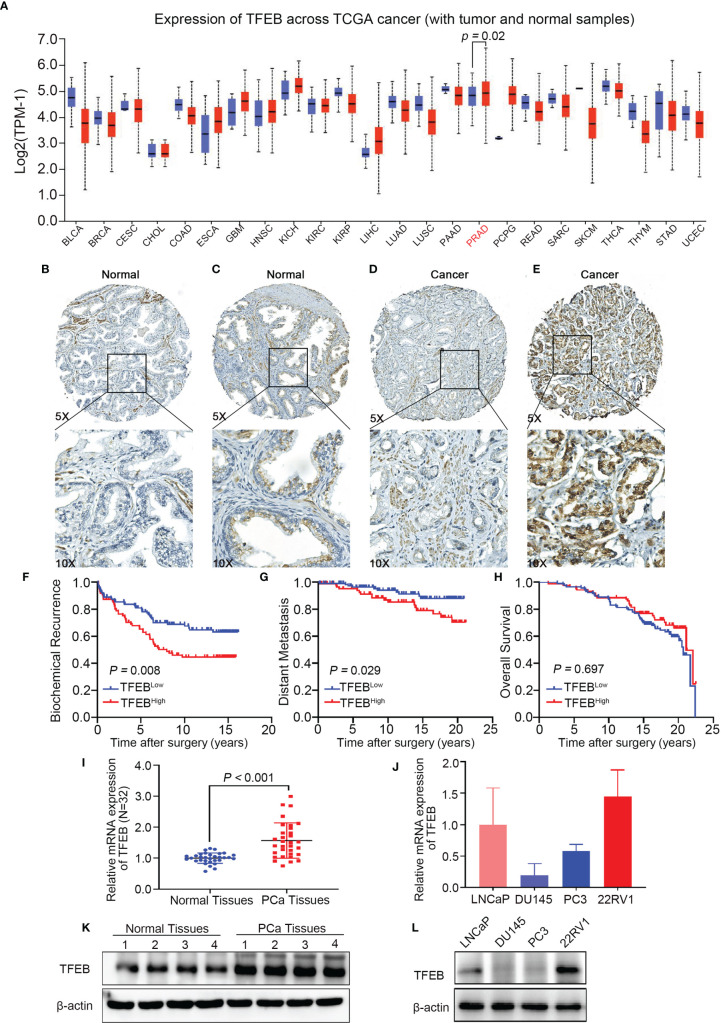
TFEB expression in prostate tissues microarray and its association with PCa progression. TFEB is often upregulated in PCa and is associated with poor outcome in PCa patients. **(A)** Bioinformatics analysis of TFEB in Pan-cancer of TCGA database. And TFEB mRNA expression was increased in PCa compared to normal tissues (*p*=0.02). **(B–D)** IHC analysis of TFEB expression in 225 PCa samples. **(B)** TFEB staining is negative in benign prostate tissue. **(C)** Moderate TFEB staining in benign prostate tissue. **(D)** The weak staining of TFEB in PCa tissues. **(E)** Strong cytoplasmic and perinuclear TFEB staining can be detected in PCa tissues. **(F–H)** Kaplan-Meier curves representing different endpoint survival of 205 patients treated with radical prostatectomy for prostate cancer stratified by TFEB status. **(F)** Biochemical recurrence-free survival (*p*=0.008). **(G)** Metastasis-free survival (*p*=0.029). **(H)** Overall death-free survival (*p*=0.697). **(I)** qRT-PCR results showed that the mRNA expression of TFEB was increased in our cohort. **(J)** Quantitative protein expression of western-blot showed TFEB expression was increased in PCa tissues compared to adjacent tissues. **(K, L)** The mRNA and protein expression of TFEB was decreased in DU145 and PC3 compared to 22RV1 and LNCaP cell lines.

To explore the role of TFEB in PCa, we confirmed TFEB mRNA expression levels in TCGA public dataset (**Figure 1A****)**. TFEB mRNA expression was found to be significantly upregulated in PCa tissues compared to normal prostate tissues. In addition, TFFB mRNA expression increased in esophageal carcinoma, glioblastoma, hepatocellular carcinoma, and kidney carcinoma. Furthermore, to investigate TFEB expression in PCa tissues, immunohistochemical staining was performed in 205 human PCa samples. TFEB showed absent or weak-to-moderate staining in the cytoplasm and perinuclear/nuclear area of benign prostatic luminal cells (**Figure 1B****)**. TFEB expression was also observed in stromal fibroblasts in a sporadic pattern (**Figures 1B, C****)**. TFEB expression in cancer cells showed diffuse cytoplasmic and perinuclear staining with sporadic nuclear expression. Absent or weak TFEB staining was found in 57.6% (118/205) of the cases (**Figure 1D**). High TFEB expression was seen in 42.4% of cases (87/205) (**Figure 1E****)**. Furthermore, postoperative metastasis occurred at a significantly higher frequency in the high TFEB expression group than the low TFEB expression group (19.5 *vs.* 8.5%, p=0.035). Meanwhile, higher preoperative PSA levels were observed in high TFEB expression cases than in cases with low TFEB expression (7.4 vs. 5.9 ng/ml, p=0.050). However, there was no significant association between TFEB expression status and other prognostic factors for PCa progression, including Gleason score (GS) (p=0.171), pT stage (p=0.425), and surgical margin (p=0.773) (**Table 1****)**.

**Table 1:** Association of TFEB expression in prostate cancer cells with clinicopathologic characteristics in 205 patients who underwent radical prostatectomy between 1993 and 1995 in Massachusetts General Hospital.

	Total	TFEB (high)	TFEB (low)	p
**Patients, no. %**	205 (100)	87 (42.4)	118 (57.6)	
**Median (IQR)**				
**Age (year)**	62 (57–67)	62 (57–67)	63 (57–67)	0.281
**PSA (ng/ml)**	6.3 (4.7–9.5)	7.4 (5.1–10.4)	5.9 (4.5–9.3)	0.050
**Prostate weight (g)**	46 (36–56)	46 (36–58)	45 (35–56)	0.606
**No. %**				
**Gleason score**				0.171
<=6	95 (46.3)	39 (44.8)	56 (47.5)	
3+4	68 (33.2)	32 (36.8)	36 (30.5)	
4+3	16 (7.8)	3 (3.5)	13 (11.0)	
>=8	26 (12.7)	13 (14.9)	13 (11.0)	
**pT stage**				0.425
<=pT2	152 (74.1)	62 (71.3)	90 (76.3)	
>=pT3	53 (25.9)	25 (28.7)	28 (23.7)	
**Surgical margin**				0.773
M−	127 (62.0)	55 (63.2)	72 (61.1)	
M+	78 (38.0)	32 (36.8)	46 (38.9)	
**Metastasis**				**0.035**
Mets−	178 (86.8)	70 (80.5)	108 (91.5)	
Mets+	27 (13.2)	17 (19.5)	10 (8.5)	

The bolded values emphasize which p value is less than 0.05.

The prognostic value of TFEB expression was examined for three different clinical outcomes, including biochemical recurrence (BCR), overall survival (OS), and distant metastasis. On univariate analysis, high expression of TFEB was significantly associated with a worse prognosis of BCR (**Figure 1F**, p=0.008) and distant metastasis (**Figure 1G**, p=0.029). According to the multivariate analysis, there was a trend for high TFEB expression being associated with a worse prognosis for BCR (p=0.065). After adjusting for independent covariates, a similar trend was observed for GS and preoperative PSA (**Table 2A**). Interestingly, the independent prognostic significance for distant metastasis (p=0.037) was sustained when adjusted by the GS (**Table 2B**). For OS, TFEB expression showed no prognostic value on univariate analysis (**Figure 1H**, p=0.697). Meanwhile, we performed mRNA and protein quantification assays to determine TFEB levels and found that TFEB was up-regulated in PCa tissues compared with normal tissues (**Figures 1I, K****)**.

**Table 2A:** Univariate and multivariate analysis of clinicopathologic factors with biochemical recurrence-free survival. Multivariate Cox regression model fitted with factors that showed significance in univariate analysis.

	Univariate			Multivariate		
	HR	95% CI	p	HR	95% CI	p
**Age (y)**	1.01	0.97–1.04	0.637	–	–	–
**PSA (ng/ml)**	1.07	1.03–1.10	**<0.001**	1.04	1.00–1.07	**0.033**
**Prostate weight (g)**	0.99	0.98–1.01	0.673	–	–	–
**Gleason score**						
**<=6**	ref					
**3+4**	1.89	1.11–3.20	**0.019**	1.86	1.04–3.32	**0.036**
**4+3**	2.17	0.94–4.98	0.068	2.04	0.86–4.84	0.105
**>=8**	6.54	3.66–11.7	**<0.001**	5.17	2.69–9.93	**<0.001**
**pT stage**						
>=pT3 *vs.* <=pT2	1.94	1.23–3.04	**0.004**	1.06	0.63–1.79	0.813
**Surgical margin**						
M (+) *vs.* M (-)	2.03	1.32–3.12	**0.001**	1.55	0.96–2.50	0.076
**TFEB IHC**						
TFEB (high) *vs.* (low)	1.78	1.16–2.74	**0.009**	1.57	0.97–2.53	0.065

The bolded values emphasize which p value is less than 0.05.

**Table 2B:** Univariate and multivariate analysis of clinicopathologic factors with metastasis-free survival. Multivariate Cox regression model fitted with factors that showed significance in univariate analysis.

	Univariate			Multivariate		
	HR	95% CI	p	HR	95% CI	p
**Age (y)**	1.01	0.94–1.07	0.903	–	–	–
**PSA (n/ml)**	1.02	0.96–1.08	0.495	–	–	–
**Prostate weight (g)**	1.00	0.98–1.02	0.754	–	–	–
**Gleason score**						
**<=6**	ref					
**3+4**	2.25	0.80–6.31	0.125	2.15	0.84–6.61	0.105
**4+3**	4.83	1.36–17.1	**0.015**	6.13	1.69–22.2	**0.006**
**>=8**	7.06	2.44–20.4	**<0.001**	6.28	2.17–18.2	**0.001**
**pT stage**						
>=pT3 *vs.* <=pT2	1.77	0.81–3.87	0.151	–	–	–
**Surgical margin**						
M (+) *vs.* M (−)	0.99	0.45–2.17	0.986	–	–	–
**TFEB IHC**						
TFEB (high) *vs.* (low)	2.33	1.06–5.08	**0.034**	2.38	1.06–5.36	**0.037**

The bolded values emphasize which p value is less than 0.05.

### Construction of Two Different Expression Pattern of TFEB in PCa Cell Lines

To further characterize the potential biological functions of TFEB in PCa, we generated two different expression patterns of TFEB according to its relative expression levels in PCa cell lines (**Figures 1J, L****)**. TFEB was stably transfected in DU145 and PC3 cell lines (DU145-TFEB or PC3-TFEB). Empty vector plasmid was transduced into two cell types, which were considered the control groups (DU145-vector or PC3-vector). Conversely, in LNCaP and 22RV1 cell lines, we established stable TFEB knockdown cells (22RV1-shTFEB and LNCaP-shTFEB) and control cells (22RV1-shNC or LNCaP-shNC). Western blotting was used to detect the expression levels of TFEB in over-expressing TFEB or TFEB knockdown cell lines **(****Supplementary Figure 1** ). Notably, TFEB protein levels in the nucleus and cytoplasm were increased in TFEB overexpressing cell lines (**Supplementary Figures 1D–F**). Moreover, in the TFEB knockdown expression cell lines, TFEB protein levels were reduced in both the cytoplasm and nucleus compared with shNC groups (**Supplementary Figures 1A–C**). TFEB is a transcription factor, which binds to the promoter region target genes to drive gene expression in nucleus (31). Therefore, evaluating TFEB protein expression in the nucleus could indicate that it might play a role in transcriptional regulation.

### TFEB Knockdown or Overexpression Influences Proliferation, Migration, and Invasion of PCa Cells

**Figure 2:**
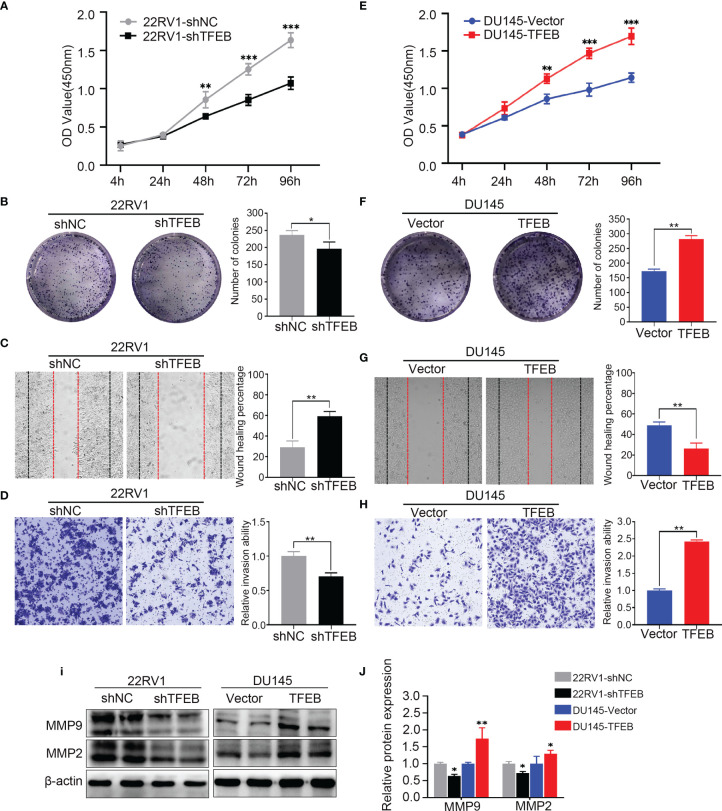
Overexpressing or knockdown expression of TFEB significantly effect PCa cell proliferation, tumorigenicity, invasion, and migration. **(A–D)** Overexpression of TFEB promotes cell proliferation, invasion, and migration *in vitro* experiment. **(E–H)** Knock down expression of TFEB inhibit cell proliferation, invasion and migration *in vitro* experiment. **(A, E)** CCK-8 assay. **(B, F)** Colony formation assay. **(C, G)** Wound healing assay; Black dashed line indicates 0-h, red dashed line indicates 48h. **(D, H)** Transwell assay; cancer cells were stained after 24h. Quantitative analysis of the colony formation assay, wound healing assay and transwell assay from panel left respectively, those assays were repeated three times. **(I, J)** Quantitative protein expression of MMP2 and MMP9 by western-blot in TFEB overexpressed or knock-down PCa cell lines. Statistical analysis was from three independent experiments and is presented as mean ± SD. *p < 0.05, **p < 0.01, ***p < 0.001 compared with control group.

Functional assays were performed to investigate the tumorigenic potential of TFEB. The results showed that the knockdown of TFEB inhibited PCa cell proliferation and colony formation ability (**Figures 2A, B****)**. Conversely, the overexpression of TFEB promoted PCa cell proliferation and colony formation (**Figures 2E, F****)**. Cancer cell invasion and migration are important events in PCa metastasis. Therefore, we evaluated the effects of TFEB on PCa cell invasion and migration. Our results showed that the silenced expression of TFEB in PCa cells inhibited cell migration and invasion ability (**Figures 2C, D****)**, whereas overexpression of TFEB resulted in increased cell migration and invasion (**Figures 2G, H****)**. Metalloproteinases are broadly recognized as being involved in cancer cell invasion and migration; thus, we analyzed the effects of TFEB on metalloproteinases by detecting the expression level of MMP2 and MMP9. Western blotting further demonstrated that MMP2 and MMP9 protein levels were increased in TFEB-overexpressing PCa cells. In contrast, TFEB-knockdown PCa cells, was associated with decreased MMP2 and MMP9 protein levels (p<0.05, **Figures 2I, J****)**.

### RNA-Sequencing Analysis Showed That TFEB Was Involved in a Lysosomal-Related Pathway in PCa

**Figure 3:**
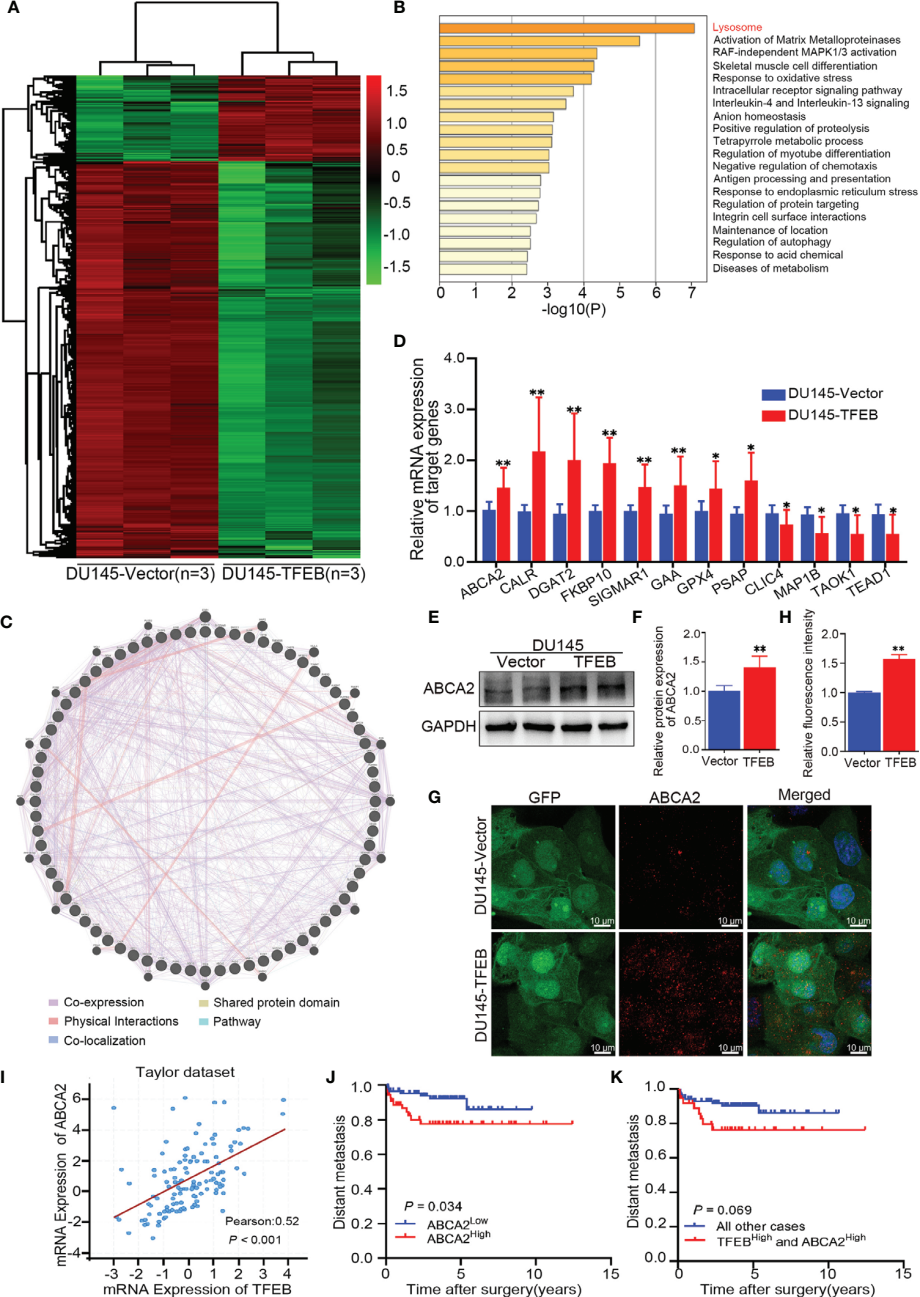
Bioinformatics analysis in TFEB-overexpressing DU145 cell lines. **(A)** The transcription differential genes heat map in DU145-TFEB *vs.* DU145-vector. The result revealed there are 1,559 differentially expressed genes (DEGs) in TFEB-overexpressing DU145 cell line compared with vector group. **(B)** Kyoto Encyclopedia of Genes and Genomes analysis showed differentially expressed genes are enrichment in lysosome biogenesis, activation of matrix metalloproteinases and autophagy pathway. **(C)** Gene-gene interaction network of DEGs predicted using the GeneMANIA online tool. **(D)** Genes of interest validated by real-time RT-PCR. **(E, F)** Western-blot verification of ABCA2 gene in TFEB-overexpressing cell lines. Quantitative analysis of the western-blot from panel **(F)**. **(G, H)** Immunofluorescence of ABCA2 in DU145cell lines. Quantitative relative fluorescence intensity of ABCA2 from **(G)**. **(I)** The correlation between TFEB and ABCA2 in Taylor dataset. **(J)** Kaplan-Meier curves representing metastasis-free endpoint survival of ABCA2 status in Taylor dataset (p=0.034). **(K)** Kaplan-Meier curves representing metastasis-free endpoint survival of combined TFEB high expression and ABCA2 high expression in Taylor dataset (p=0.069). Statistical analysis was from three independent experiments and is presented as mean ± SD. *p < 0.05, **p < 0.01 compared with control group.

Recent studies have shown that TFEB plays different biological roles in different tumors or diseases; however, its biological function in PCa has not yet been fully elucidated. Therefore, we utilized an RNA-sequencing strategy to investigate whether TFEB had a role in PCa. DU145-vector and DU145-TFEB cell lines were used for analyses by RNA-sequencing. Compared to the control DU145-vector cell line, and we found that many genes were significantly up-regulated in the DU145-TFEB group (**Figure 3A****)**. Next, using Metascape website tools (http://metascape.org), we selected those differentially expressed genes (DEGs) to perform biological annotation and functional enrichment analysis in order to explore the role of TFEB in PCa. As shown in **Figure 3B**, we found that upregulated DEGs were enriched in the lysosome-related pathways, which meant part of these genes were involved in lysosome biogenesis and lysosomal enzyme activity. Besides, we observed that matrix metalloproteinases and autophagy pathways were also enriched (**Figure 3B****)**. We then used the GENEMANIA analysis tool (http://genemania.org/) to explore the functions or interactions between those genes to understand their biological roles and possible regulation by TFEB. Most of these genes presented a co-expression pattern, which meant they might share a similar regulatory network that participates in lysosome activation (**Figure 3C**). Of note, part of upregulated DEGs belonged to the CLEAR (Coordinated Lysosomal Expression and Regulation) gene network regulated by TFEB and involved in lysosomal biogenesis, proliferation, proteostasis, and acidification. Furthermore, to verify the RNA-seq results, we selected many genes related to the lysosome for PCR and western blotting verification. ABCA2, CALR, DGAT2, FKBP10, SIGMAR1, GAA, GPX4, and PSAP genes mRNA levels showed a significant increase in the DU145-TFEB cell line than in the DU145-vector controls (**Figure 3D****)**. CLCI4, MAP1B, TAOK1, and TEAD1 were down-regulated in DU145-TFEB overexpressing cells. At the protein level, a significant increase in ABCA2 protein, a membrane protein located on the lysosome, was observed in the TFEB over-expressing cell lines compared with the control groups (**Figures 3E, F****)**. Cellular immunofluorescence showed ABCA2 was mainly expressed in the cytoplasm and upregulated in TFEB over-expressing cell lines compared with the vector groups (**Figures 3G, H****)**. Furthermore, we detected ABCA2 protein expression in the TFEB knockdown cell lines and found that ABCA2 expression was significantly decreased in the TFEB knockdown groups **(****Supplementary Figure 2****)**.

ABCA2 is located on the lysosome surface and mainly transports various molecules across the lysosome and cytoplasm, which is important for lysosomal function. By analyzing the Taylor dataset (20), we found a positive correlation between TFEB and ABCA2 in the Taylor dataset (p<0.001, **Figure 3I**). Furthermore, Kaplan-Meier curves indicated worse metastasis outcomes in PCa patients with high expression of ABCA2 (p=0.034, **Figure 3J**). In addition, we evaluated the prognostic value of the combined expression of TFEB and ABCA2, and observed a trend for worse prognosis in terms of metastasis outcome albeit the trend was not statistically significant (p=0.069, **Figure 3K****)**.

### TFEB Regulated Lysosomal Biogenesis in PCa Cells

**Figure 4:**
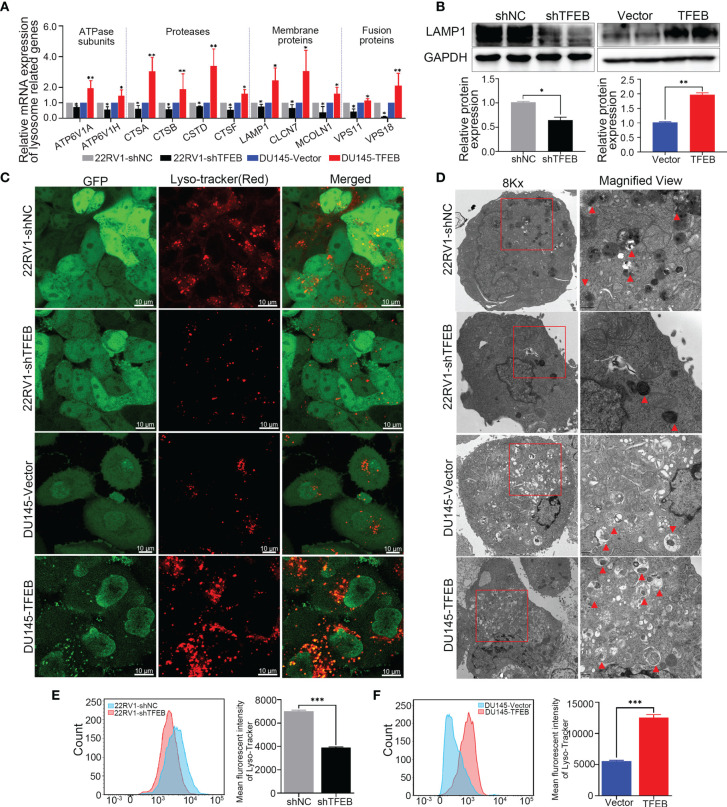
Overexpression of TFEB induced lysosome biogenesis in PCa cell. Knockdown TFEB expression decreased lysosome biogenesis in PCa. **(A)** Validation of lysosome related genes by qRT-PCR in different PCa cell lines. **(B)** Validation of lysosome marker protein LAMP1 by western-blot in different PCa cell lines. Quantitative analysis of the western-blot shows LAMP1 expressed significantly differently in TFEB-overexpressing or TFEB knockdown cell lines. **(C)** PCa cells were treated with Lyso-Tracker Red DND-99 (50 nM) for 45 min. **(D)** Transmission electron microscope (TEM) image of lysosomes or autophagosomes in PCa cells. **(E, F)** The quantitative measurement of Lyso-Tracker Red DND-99 (50 nM) was performed by flow cytometry. Statistical analysis was from three independent experiments and is presented as mean ± SD. *p < 0.05, **p < 0.01, ***p < 0.001 compared with control group.

To examine the effects of TFEB on lysosomal biogenesis and function, several assays were used to determine its roles in lysosomal function in different cells. First, as showed in **Figure 4A**, lysosomal-related genes were upregulated in DU145-TFEB and down-regulated in 22RV1-shTFEB cells, and included ATP subunits genes (ATP6V1A and ATP6V1H), proteases genes (CTSA, CTSB, CTSD, and CTSF), membrane genes (LAMP1, CLCN7, and MCOLN1), and fusion genes (VPS11 and VPS18). Second, LAMP1, a membrane protein considered to be a lysosomal marker, was used to assess the intracellular lysosome number. *In vitro* experiments showed that TFEB could influence LAMP1 expression in TFEB transfected cell lines (**Figure 4B**). To determine how TFEB affects lysosome biogenesis and function, Lyso-Tracker Red (DND-99) was used to assess the number and morphology of live lysosomes in different PCa cell lines. As shown in **Figure 4C**, the number of lysosomes in the cytoplasm and fluorescence intensity was increased in DU145-TFEB transfected cells compared with DU145-vector control cells, and was decreased in 22RV1-shTFEB compared with 22RV1-shNC cells. Furthermore, there was no significant difference in lysosomal morphology across any of the groups. Based on the electron microscopy findings, we found that the number of lysosomes was increased in the TFEB over-expressing cell line and was decreased in the TFEB knockdown cell line compared with untransfected controls (**Figure 4D**). Third, flow cytometry was used to quantitate lysosome numbers stained by Lyso-Tracker Red. We found decreased lysosome numbers in the TFEB knockdown PCa cells compared to the shNC group (**Figure 4E**); while, an increase in lysosome numbers was observed in the TFEB over-expressing cell line (**Figure 4F****)**.

### Overexpression of TFEB Promoted Xenograft Tumor Growth in PCa

**Figure 5:**
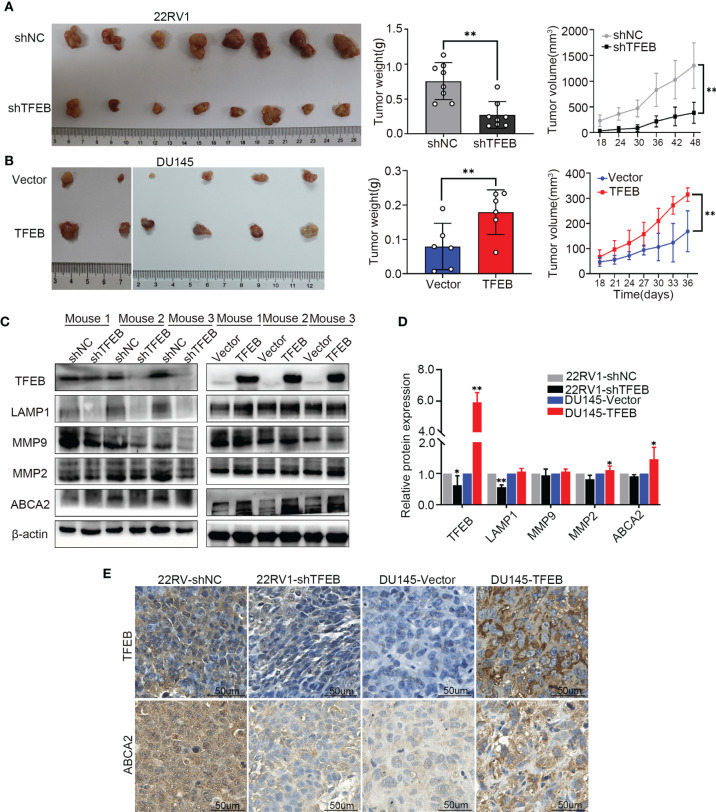
The TFEB xenograft tumor model and the validation of MMP9, MMP2 and ABCA2. **(A)** Knock-down TFEB expression inhibit PCa growth in 22RV1 xenograft tumor. The upper column is the control group (shNC), and the bottom line is the TFEB knockdown group (shTFEB). The curves of tumor volume and weight are also shown at right panel. **(B)** Overexpression of TFEB promoted PCa growth in DU145 xenograft model. The upper column is the control group (Vector), and the bottom line is the TFEB overexpression group (TFEB). The curves of tumor volume and weight are also shown at right panel. **(C, D)** Validation of TFEB, LAMP1, MMP9, MMP2, and ABCA2 protein expression by western-blot. Quantitative analysis of the western-blot from panel **(C)**. **(E)** Immunohistochemistry of TFEB and ABCA2 in TFEB knockdown and TFEB overexpressing xenograft tumor samples. Statistical analysis was from three independent experiments and is presented as mean ± SD. *p < 0.05, **p < 0.01 compared with control group.

To assess the ability of TFEB to affecting tumorigenicity of PCa cells *in vivo*. 22RV1-shTFEB and 22RV1-shNC cells were subcutaneously injected into the left and right dorsal flanks of eight nude mice (n=8). Further, DU145-vector and DU145-TFEB were subcutaneously injected into the left and right dorsal sides six nude mice per condition (n=6). The xenograft tumors were checked every 3 days or 6 days; then, the mice were sacrificed at the specified time. Furthermore, the xenograft tumors samples were collected for further study. Two DU145 nude mice were sacrificed in advance because they were in senescence and exhibited poor conditions. Compared with the control groups, tumors formed following TFEB knockdown were markedly reduced (p < 0.05, **Figure 5A****)**, and tumors in xenografts containing TFEB-overexpressing DU145 cells were significantly larger (p < 0.05, **Figure 5B****)**.

In the xenograft tumors, we found that TFEB was highly expressed in the DU145-TFEB group compared to the DU145-vector group, which revealed the effects of TFEB overexpression (**Figures 5C, D****)**. Moreover, TFEB expression was decreased in the 22RV1-shTFEB group compared to the 22RV1-shNC group. In addition, we found that LAMP1, a lysosome marker protein, was decreased in the 22RV1-shTFEB group and was increased in the DU145-TFEB group. By investigating metalloproteinase expression to assess invasion or migration ability of TFEB *in vivo*, we found that MMP9 and MMP2 protein expression were increased compared with the vector-transfected group. Furthermore, MMP9 and MMP2 protein expression were decreased in the TFEB knockdown group (**Figures 5C, D****)**. The above results were consistent with the *in vitro* experiments. Next, we detected the protein expression of potential downstream genes, such as ABCA2. In the TFEB knockdown xenograft tumor groups, ABCA2 protein expression was decreased, while an increased expression was observed in TFEB-overexpressing xenograft tumor groups compared to vector groups (**Figures 5C, D****)**. The IHC assays revealed that the expression of TFEB was decreased in 22RV1-shTFEB xenograft tumor samples and was increased in the DU145-TFEB xenograft tumor samples compared to the control group Furthermore, ABCA2 expression in the cytoplasm was increased in TFEB-overexpressing xenograft tumor samples and was decreased in TFEB knockdown xenograft tumors (**Figure 5E****)**.

### TFEB Regulated ABCA2 Expression to Influence Invasion and Migration of PCa

**Figure 6:**
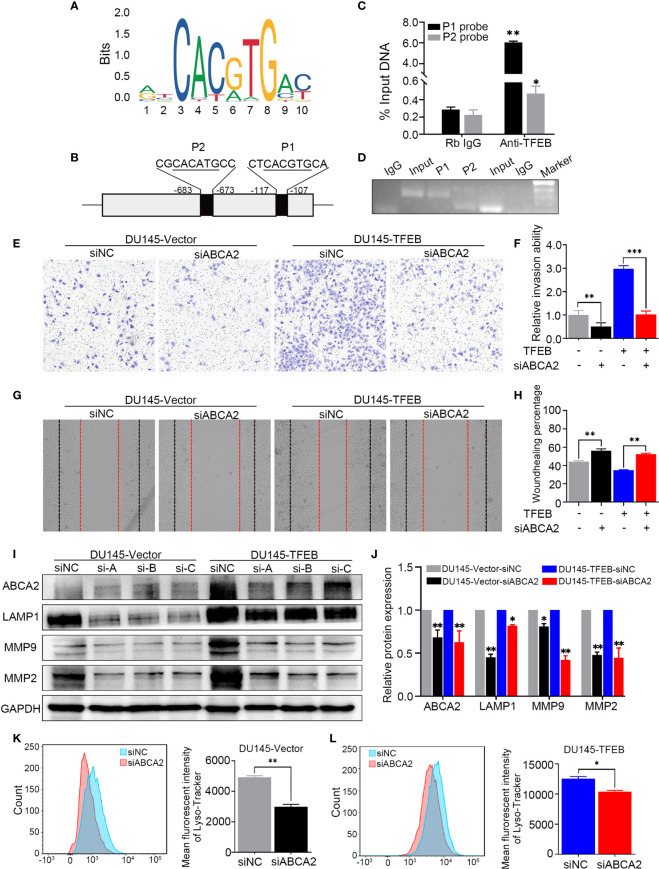
TFEB binding ABCA2 promoter to regulate its expression to involve PCa cell invasion and migration. **(A)** The binding motif of TFEB were provide from website. **(B)** The potential binding site of ABCA2 promoter. Mismatch rate is less than 1%. **(C)** Validation of the DNA fragment pulled down with TFEB chip-level antibody by qRT-PCR. DNA fragment were obtained from CUT&RUN assay and purified by DNA extraction kit. Rb IgG as a negative control. Anti-TFEB as an experimental group. **(D)** The DNA fragment product from qRT-PCR was validated by nucleic acid electrophoresis. The length of input, P1 and P2 mainly between 60 to 120 bp. **(E, F)** Transwell assay showed silenced ABCA2 expression inhibited PCa cell invasion. Cancer cells were stained after 24h. **(G, H)** Woundhealing assay showed silenced ABCA2 expression inhibited PCa cell migration after 48h culture. **(I, J)** Validation of ABCA2 LAMP1, MMP9, and MMP2 protein expression by western-blot after ABCA2 gene silenced. Quantitative analysis of the western-blot from **(I)**. **(K, L)** DU145-vector and DU145-TFEB cell line were silenced ABCA2 for 72h and then treated with LysoTracker Red DND-99 (50 nM) for 45 min. Note: Statistical analysis was from three independent experiments and is presented as mean ± SD. *p < 0.05, **p < 0.01, ***p < 0.001 compared with control group.

ABCA2, a member of the superfamily of ATP-binding cassette transporters, is located on the lysosome membrane and mainly transports lipids and chemotherapy drugs. In this study, our findings revealed that TFEB influenced ABCA2 expression both in *in vivo* and *in vitro* experiments; however, the mechanisms regulating TFEB and ABCA2 expression not been elucidated. To mechanistically understand how TFEB regulated ABCA2 expression, the Cleavage Under Targets and Release Using Nuclease (CUT&RUN) assay was used to analyze the potential regulatory mechanism involved. First, using the bioinformatics website tool to analyze the potential binding sites of the promoter region, we searched for potential binding sites according to the TFEB motif sequence (**Figure 6A****)** and identified two different potential binding sites, P1 and P2 (**Figure 6B****)**. Using the CUT&RUN assay kit, we determined the TFEB binding sequence in the DU145-TFEB cell line, and the results showed that TFEB indeed bound to the promoter region of ABCA2 to increase the expression of ABCA2 in DU145-TFEB transfected cells compared with DU145-vector controls. P1 and P2 expression were higher than the IgG-treated negative control group (p<0.05). The 2^−△CT^ value of P1 was higher than the P2 (**Figure 6C****)**, which meant TFEB might have a higher binding capacity at P1 than P2. Furthermore, the nucleic acid electrophoresis findings revealed that the size of the two binding site products were between 60 and 150 bp, which was compliant with the standard of CUT&RUN assay (**Figure 6D****)**. Furthermore, to determine whether TFEB might regulate ABCA2 activity during lysosomal biogenesis, which may ultimately influence PCa cell invasion and migration, we silenced ABCA2 expression in DU145-TFEB and DU145-vector cell lines to explore its influence in PCa invasion, migration, and lysosomal biogenesis. As shown in **Figures 6E** and **F**, silencing ABCA2 in DU145 cell lines resulted in decreased invasion ability (p<0.001). In addition, silencing ABCA2 expression reduced cancer cell migration ability in PCa (p<0.001, **Figures 6G, H****)**. Furthermore, western blotting showed that, after silencing ABCA2 in DU145 cell lines, ABCA2, LAMP1 MMP9, and MMP2 protein expression were decreased (p<0.05, **Figures 6I, J****)**. In addition, we also detected the number of lysosomes by flow cytometry and observed reduced numbers after silencing ABCA2 expression in PCa cell lines (p<0.05, **Figures 6K, L****)**.

## Discussion

PCa is regarded as one of the most frequently diagnosed types of malignancy worldwide (1). In the past decades, PSA has often been used to diagnose early staged PCa patients. At present, many novel markers have appeared for accurate diagnosis in the early stages and to suggest precise treatment in PCa (32), especially TME-related biomarkers like TRIB1 (33). However, because of differentiation of the biological heterogeneity in PCa, currently available biomarkers and indexes cannot precisely estimate the risk in aggressive PCa patients, who may eventually experience BCR, develop castration-resistant prostate cancer, or metastasis. It is crucial to identify novel biomarkers for a more accurate diagnosis of PCa.

TFEB is the second-most characterized member of the MiT family, and shares DNA binding, HLH, and Zip regions (6). Previous cancer-related studies have demonstrated that TFEB is dysregulated in many cancers, Sounak *et al*. have suggested that TFEB is overexpressed in TFEB-rearranged renal cell carcinomas (RCC) and is associated with aggressive biological behavior: TFEB amplification in RCC patients has been associated with poor outcome compared to other types of RCC (34). A study reported that TFEB is a master regulator of tumor-associated macrophages in the breast TME, downregulation of TFEB-induced macrophage polarization and a tumor-promoting phenotype. However, it has not been determined how TFEB exerts its role in PCa. In this study, we revealed for the first time that TFEB expression was upregulated in PCa tissue samples at both the mRNA and protein levels. Moreover, high TFEB expression correlated with higher preoperative PSA levels and metastasis status. Patients with overexpressed TFEB tended to have poor prognosis.

To further validate the role of TFEB in PCa, we used PCa cell lines to perform *in vivo* and *in vitro* experiments. Lentivirus-mediated knockdown of TFEB in PCa cells had lower invasiveness and migration capabilities. Further, overexpression of TFEB significantly promoted PCa cell proliferation, invasion, and migration. Our results were consistent with Lu et al., who had clarified that overexpression of TFEB significantly reduced the number of apoptotic cells in vascular smooth muscle, and TFEB knockout caused an increase in the number of apoptotic cells. It might due to TFEB directly regulate the expression of anti-apoptosis gene BCL2 (35). However, we did not explore more mechanism about the regulation of apoptosis by TFEB. Of note, TFEB-overexpressing xenograft tumors exhibited an increase in tumor weight and volume, while knocked-down TFEB expression resulted in slower growth in xenograft tumors. Nevertheless, our objective was to determine how TFEB affected PCa. Thus, we used RNA-sequencing and bioinformatics analysis to identify 1559 differentially expressed genes in TFEB over-expressing cell lines compared to the vector control group. Most upregulated genes showed a co-expression pattern and were enriched in a lysosome-related pathway. Furthermore, we found that overexpressing TFEB in the PCa cell line induced an increased number of lysosomes and higher lysosomal enzyme activity. Those findings were consistent with other studies performed by Sardiello et al., who reported that TFEB could regulate coordinated lysosomal expression and the expression of (CLEAR) network genes, as well as promote lysosomal biogenesis in degenerative storage diseases (8).

Growing evidence has revealed that the lysosome acts as a metabolic or growth regulator in many kinds of cancer, and its activation could affect cancer cell proliferation, invasion, and migration (36). Lysosome-related pathway activation may play a dual role in cancer development. Lysosomes could induce apoptosis by releasing cathepsin and cleavage of Bid, which is a proapoptotic protein, and may generate Bax-mediated release of cytochrome c (37). Besides, lysosomes can fuse with autophagosomes to degrade damaged organelles and produce amino acids, sugars, lipids, and nucleotides to support cancer cell growth (15). LAMP1, as a marker of the lysosome has been used to assess the number of lysosomes. Downregulation of LAMP1 inhibited PCa cell proliferation, invasion, and migration ability (38). It is well known that effective lysosomal functions are essential for advanced cancer cells. Lysosomes can fuse with autophagosomes to degrade damaged organelles and provide energy and factors able to support cancer cell proliferation, invasion, and migration (39). Cancer progression and metastasis are related to conspicuous lysosome changes, including lysosome numbers, volume, and lysosomal enzyme activity. Kundu et al.. found that TMEM106B can promote lung cancer cell invasion and metastasis through TFEB-mediated lysosome biogenesis (10). High expression levels of lysosomal cathepsins are frequently associated with metastasis and poor prognosis (36). Consequently, in our study we demonstrated that TFEB overexpression induced lysosome synthesis and promoted cancer cell proliferation, invasion, and migration in PCa. Based on these results, we speculated that the increased lysosomal biogenesis fueled PCa cells malignant phenotype through degrading damaged organelles for providing energy and material basis.

Matrix metalloproteinases (MMPs) are essential to cancer cell invasion and metastasis, especially MMP2 and MMP9 (40). MMPs will be released by tumor cells into the TME to influence stroma cell growth and degrade the extracellular matrix, which results in the induction of cancer cell invasion and tumor spread. Many studies have used MMP9 and MMP2 as indicators for evaluating cancer cell invasion and migration ability (41, 42). In our study, we found that MMP2 and MMP9 were upregulated in the TFEB over-expressing cell line and were downregulated in the TFEB knockdown cell line group *in vivo* and *in vitro*. It has also been reported that lysosome activation can release active protease cathepsin B to simulate MMP9 activity by cleaving its endogenous inhibitor TIMP-1 (43), which might provide additional evidence to support our results. Therefore, TFEB might regulated MMP2 and MMP9 expression through lysosomal biogenesis in the tumor environment. Moreover, we have identified a novel target gene, ABCA2, associated with lysosomal biogenesis by RNA-sequencing, which might affect MMP9 and MMP2 expression in the TME.

ABCA2 is the second member of the superfamily of ABC transporters and is located on the lysosome surface (44). ABCA2 is a uniport carrier of various substrates across the cytoplasm into the lysosome, including lipid and chemotherapy drugs, and thus might affect lysosomal biogenesis. Aberuyi et al. have reported that ABCA2 is upregulated in acute lymphoblastic leukemia and may contribute to multidrug resistance by transporting drugs localized in the cytoplasm into the lysosome for degradation (45). Using Abca2 knockout mice, Kenneth et al. found that ABCA2-deficiency inhibited PCa metastatic progression and altered metastatic localization in the TME (46). In our study, we found that mRNA expression of ABCA2 was up-regulated in TFEB over-expressing cell lines and was positively associated with TFEB in the Taylor dataset. In addition, patients with high ABCA2 expression exhibited a worse metastasis prognosis in PCa. Furthermore, the prognostic value of the combination of ABCA2 and TFEB expression showed a trend for poor outcomes in PCa. In this study, we used the novel CUT&RUN assay, which is a method used to analyze protein interactions with DNA, to detect the relationship between ABCA2 and TFEB (45). Using this approach, we defined the anti-TFEB binding DNA sequence in PCa cells, and qRT-PCR revealed that TFEB had binding sites located within the ABCA2 promoter region, one of which showed a higher binding capacity (P1>P2). Moreover, rescue assays indicated that silenced ABCA2 in TFEB over-expressing or the vector control group reduced PCa cell invasion and migration ability, and MMP9 and MMP2 protein expression were also significantly decreased. Conversely, LAMP1 protein expression and the fluorescence intensity of Lyso-Tracker DND-99 was decrease when ABCA2 was silenced, which meant lysosomal biogenesis was also reduced in PCa. These results illustrated that TFEB promoted lysosomal biogenesis by regulating ABCA2 expression, and induced MMP9 and MMP2 upregulation in the TME to enhance PCa cell invasion and metastasis.

Our results revealed a novel mechanism underlying PCa progression. We showed that TFEB expression was increased in PCa tissues, and played a role in the progression of PCa by influencing ABCA2 activity in cancer-related lysosome biogenesis, which might contribute to patient’s poor clinical prognosis. Further research is needed to uncover the underlying role of ABCA2 in lysosomal biogenesis and PCa progression. Taken together, TFEB might serve as a novel therapeutic and prognostic target in PCa patients.

## Data Availability Statement

The datasets presented in this study can be found in online repositories. The names of the repository/repositories and accession number(s) can be found below: GEO, GSE163761.

## Ethics Statement

The studies involving human participants were reviewed and approved by The human study ethics committees at Guangzhou First People’s Hospital. The patients/participants provided their written informed consent to participate in this study. The animal study was reviewed and approved by Research Ethics Committee of Guangzhou Medical University.

## Author Contributions

WZ and GZ supervised the whole project and study, participated in study design and coordination. XZ, YZ, and SW collected and analyzed the data. C-LW and BW analyzed and evaluated the IHC of TMA.YC, JY, YD, YF, RL, SC, and ZZ performed the experiments. WZ wrote the manuscript. GZ revised this manuscript. All authors contributed to the article and approved the submitted version.

## Funding

This work was supported by grants from National Natural Science Foundation of China (82072813, 81874099), Guangzhou Municipal Science and Technology Project (201803040001), The Science Foundation of Guangzhou First People’s Hospital (Q2019020), China Postdoctoral Science Foundation (2020M682666), Guangdong Medical Science Research Project (A2020544).

## Conflict of Interest

The authors declare that the research was conducted in the absence of any commercial or financial relationships that could be construed as a potential conflict of interest.
