# Corrigendum: TFEB Promotes Prostate Cancer Progression *via* Regulating ABCA2-Dependent Lysosomal Biogenesis

**DOI:** 10.3389/fonc.2021.750277

**Published:** 2021-08-30

**Authors:** Xuejin Zhu, Yangjia Zhuo, Shulin Wu, Yanfei Chen, Jianheng Ye, Yulin Deng, Yuanfa Feng, Ren Liu, Shanghua Cai, Zhihao Zou, Bin Wang, Chin-Lee Wu, Guohua Zeng, Weide Zhong

**Affiliations:** ^1^Department of Urology and Guangdong Key Laboratory of Urology, The First Affiliated Hospital of Guangzhou Medical University, Guangzhou, China; ^2^Department of Urology, Guangdong Key Laboratory of Clinical Molecular Medicine and Diagnostics, Guangzhou First People’s Hospital, Guangzhou Medical University, Guangzhou, China; ^3^Department of Urology, Guangdong Key Laboratory of Clinical Molecular Medicine and Diagnostics, Guangzhou First People’s Hospital, School of Medicine, South China University of Technology, Guangzhou, China; ^4^Department of Urology, Massachusetts General Hospital, Harvard Medical School, Boston, MA, United States; ^5^Department of Pathology, Massachusetts General Hospital, Harvard Medical School, Boston, MA, United States; ^6^Department of Urology, Affiliated Cancer Hospital & Institute of Guangzhou Medical University, Guangzhou Medical University, Guangzhou, China

**Keywords:** TFEB, ABCA2, prostate cancer, tumor microenvironment, lysosomal biogenesis, biochemical recurrence, metastasis

In the original article, there was a mistake in ***Figure 6*** as published. **Figure 6 was misplaced and needs to be corrected.** The corrected ***Figure 6*** appears below.

**Figure 6:**
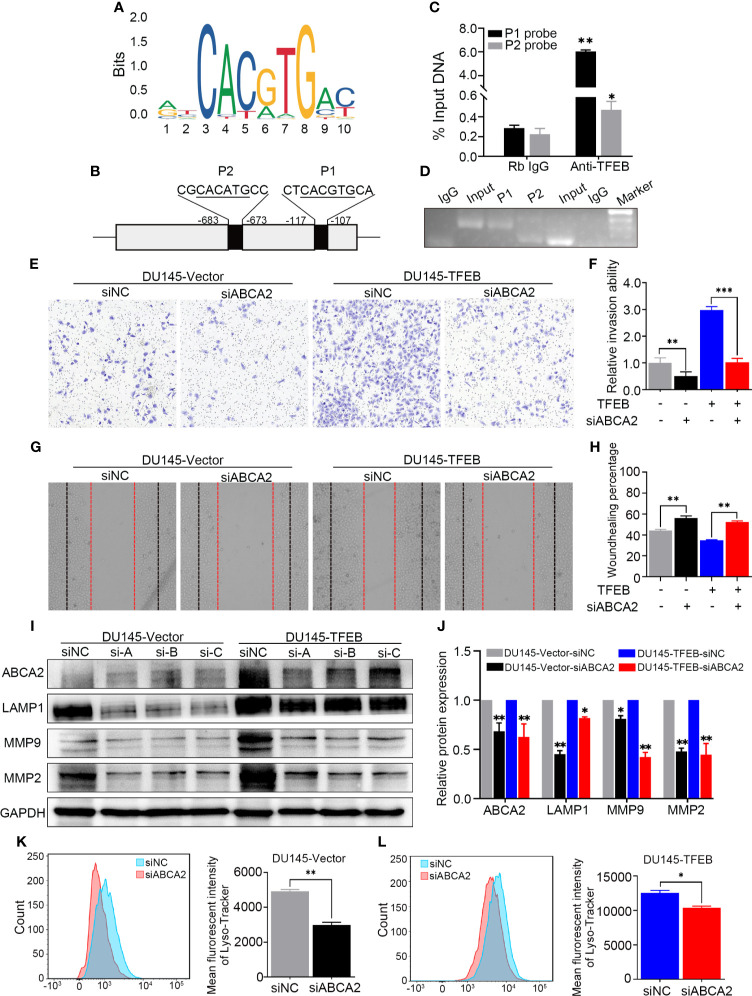
TFEB binding ABCA2 promoter to regulate its expression to involve PCa cell invasion and migration. **(A)** The binding motif of TFEB were provide from website. **(B)** The potential binding site of ABCA2 promoter. Mismatch rate is less than 1%. **(C)** Validation of the DNA fragment pulled down with TFEB chip-level antibody by qRT-PCR. DNA fragment were obtained from CUT&RUN assay and purified by DNA extraction kit. Rb IgG as a negative control. Anti-TFEB as an experimental group. **(D)** The DNA fragment product from qRT-PCR was validated by nucleic acid electrophoresis. The length of input, P1 and P2 mainly between 60 to 120 bp. **(E, F)** Transwell assay showed silenced ABCA2 expression inhibited PCa cell invasion. Cancer cells were stained after 24h. **(G, H)** Woundhealing assay showed silenced ABCA2 expression inhibited PCa cell migration after 48h culture. **(I, J)** Validation of ABCA2 LAMP1, MMP9, and MMP2 protein expression by western-blot after ABCA2 gene silenced. Quantitative analysis of the western-blot from **(I)**. **(K, L)** DU145-vector and DU145-TFEB cell line were silenced ABCA2 for 72h and then treated with LysoTracker Red DND-99 (50 nM) for 45 min. Note: Statistical analysis was from three independent experiments and is presented as mean ± SD. *p < 0.05, **p < 0.01, ***p < 0.001 compared with control group.

In the published article, there was an error in affiliation **1, 2**. Instead of “

^1^Department of Urology, Guangdong Key Laboratory of Clinical Molecular Medicine and Diagnostics, Guangzhou First People’s Hospital, Guangzhou Medical University, Guangzhou, China

^2^Department of Urology and Guangdong Key Laboratory of Urology, The First Affiliated Hospital of Guangzhou Medical University, Guangzhou, China”,

it should be “

^1^Department of Urology and Guangdong Key Laboratory of Urology, The First Affiliated Hospital of Guangzhou Medical University, Guangzhou, China

^2^Department of Urology, Guangdong Key Laboratory of Clinical Molecular Medicine and Diagnostics, Guangzhou First People’s Hospital, Guangzhou Medical University, Guangzhou, China”.

In the published article, there was an error regarding the affiliation**s** for Weide Zhong. As well as having affiliation(s) **1, 3,** they should also have, **2**.

The authors apologize for these errors and state that this does not change the scientific conclusions of the article in any way. The original article has been updated.

## Publisher’s Note

All claims expressed in this article are solely those of the authors and do not necessarily represent those of their affiliated organizations, or those of the publisher, the editors and the reviewers. Any product that may be evaluated in this article, or claim that may be made by its manufacturer, is not guaranteed or endorsed by the publisher.

