# Supplementary material for: TFEB Promotes Prostate Cancer Progression via Regulating ABCA2-Dependent Lysosomal Biogenesis
Source: Front Oncol. 2021 Mar 1;11:632524. doi: 10.3389/fonc.2021.632524 (PMC7959325; doi:10.3389/fonc.2021.632524)
Supplement: Supplementary file 1 [file DataSheet_1.pdf]

Supplementary Material

Supplementary Materials and Methods

Patients and tissue samples

Two hundred and five consecutive patients with PCa who underwent radical prostatectomy at a single hospital from 1993 to 1995 were included in this study with the approval of the Institutional Review Board. Patients who received adjuvant or neoadjuvant hormonal or radiation treatment prior to cancer recurrence were excluded. All hematoxylin–eosin (H&E)-stained sections from each case were reviewed, and the Gleason score (GS) was reassigned based on the current grading recommendation from the International Society of Urological Pathology. The tissue microarray (TMA) manufacturing process was described earlier. Relevant clinicopathological data for each patient included age, preoperative PSA, GS, pathological T stage (pT), surgical margin (M), biochemical recurrence (BCR), postoperative metastasis and overall survival (OS). Postoperative BCR was defined as a post-nadir detectable serum PSA level of ≥0.2 ng/ml, followed by a confirmatory value. Metastasis was defined by the diagnosis of prostate cancer recurrence in a lymph node or at a distant site by clinical impression and radiographic evidence. Information on death was taken from death certificates and physician correspondence. The time intervals between initial surgery and different endpoints (BCR, metastasis and OS) were defined separately. The median follow-up time among all patients was 16.4 years (IQR 14.4-19.4). During the follow-up period, 40.5% of patients had BCR, 13.2% of patients developed metastases, and 27.8% of patients died from all causes.

Real-time PCR

All genes primers were designed by Oligo 7 software (v7.56). Those primers were evaluated with high scores using Oligo 7 software and would be choose in this study. List of human primers for RT-PCR.

Gene Name	Primer Sequence
TFEB F	CAGATGCCCAACACGCTACC
TFEB R	TGTCTTTCTTCTGCCGCTCC
ABCA2 F	AGAAGTGCATCGCGTCCCTC
ABCA2 R	GCCACCTCATACAGCGCGAAG
CALR F	ATCAACAAGGACATCCGTTGC
CALR R	CTCATCCCAGTCTTCCGGTT
DGAT2 F	CGGGACACCATAGACTATTTGCT
DGAT2 R	GCCCTCCTCGAAGATCACCT
FKBP10 F	TCCAGCTACTCCCGCAACCAC
FKBP10 R	ATGGTAATTCTCCGGCGTTCCC
SIGMAR1 F	CCTTCTGCACGCCTCGCTGTCC
SIGMAR1 R	ACCTCACTTTTGGTGGTGCCCTC
GAA F	GCTACTCCTCCACCGCTATCACC

GAA R	CCATGTAGTCCAGGTCGTTCCAC
GPX4 F	TGGCCTTCCCGTGTAACCAGT
GPX4 R	CCCGTTCACGCAGATCTTGCT
PSAP F	CCATTAAGAAGCACGAGGTCCC
PSAP R	CCTCCTGGCACTCTTCCGAC
CLIC4 F	ACGTAAATTTCTGGATGGCAA
CLIC4 R	GTATTGGTGAACTCGTCCCT
MAP1B F	GTCCCCTTCTCAGGAATTCGT
MAP1B R	CCTTCAGAAAATCCGTTGAGC
TAOK1 F	AGTGCCTTATATCACATAGCC
TAOK1 R	AGTTGCGAAAATAATCAGACCA
TEAD1 F	TGCCAACCATTCTTACAGTGACC
TEAD1 R	CAGCCCAGAATTTTACGAGGA
ACTB F	ACCCTGAAGTACCCCATCGAG
ACTB R	AGCACAGCCTGGATAGCAAC
GAPDH F	ACAGCCTCAAGATCATCAGC
GAPDH R	GGTCATGAGTCCTTCCACGAT
ATP6V1A F	GTACTTCGCACTGGTAAACCC
ATP6V1A R	AGTGATATGACTACCAACCCGTA
ATP6V1H F	ATGACCAAAATGGATATCCGA
ATP6V1H R	CAAAGAAAATGCTAACACGCTGA
CTSA F	TGGGCTTCTCCTACTCCGAT
CTSA R	CAGCATAGCTCTCCCCGGTCA
CTSB F	ATGTAGGGTGCAGACCGTACT
CTSB R	GTGTGCCATTCTCCACTCCC
CTSD F	CGCTGCACAAGTTCACGTCCA
CTSD R	CCCGATGCCAATCTCCCCGTA
CTSF F	AGCCCAAGTCAGCCTTCACTC
CTSF R	AGCCATCTTCACAGGCAAGTCC
LAMP1 F	CACTTCTGACCCCAGTCTCG
LAMP1 R	TCACGTTGTTCATGTGGACCT
CLCN7 F	CCATGATCTCCACGTTCACCCT
CLCN7 R	CCAGCACGGCCTCAATCACC
MCOLN1 F	CCGCTCACTCCTTCGAGGCTT
MCOLN1 R	ACCCACACCAGCAGCGTCGAG
VPS11 F	CTGCTCAGCCCTAAGTGACC
VPS11 R	ACATCCTCAAAGACGGTGCT
VPS18 F	CAAGGCAAATGAGCCCAACCAC
VPS18 R	CCCAGTGCCTTGTTCCAACCC
ABCA2 P1 F	ACAAGCCCGAGCAACATAGCG
ABCA2 P1 R	GCCTCAGCCCTCCAAGTAGC

ABCA2 P2 F	AGCTGGATACCATTGACAACGC
ABCA2 P2 R	CCACCGCTCACCTTGGACA
