# Supplementary material for: TFEB Promotes Prostate Cancer Progression via Regulating ABCA2-Dependent Lysosomal Biogenesis
Source: Front Oncol. 2021 Mar 1;11:632524. doi: 10.3389/fonc.2021.632524 (PMC7959325; doi:10.3389/fonc.2021.632524)
Supplement: Supplementary file 2 [file DataSheet_2.pdf]

Supplementary Figure 1

Supplementary Figure 1

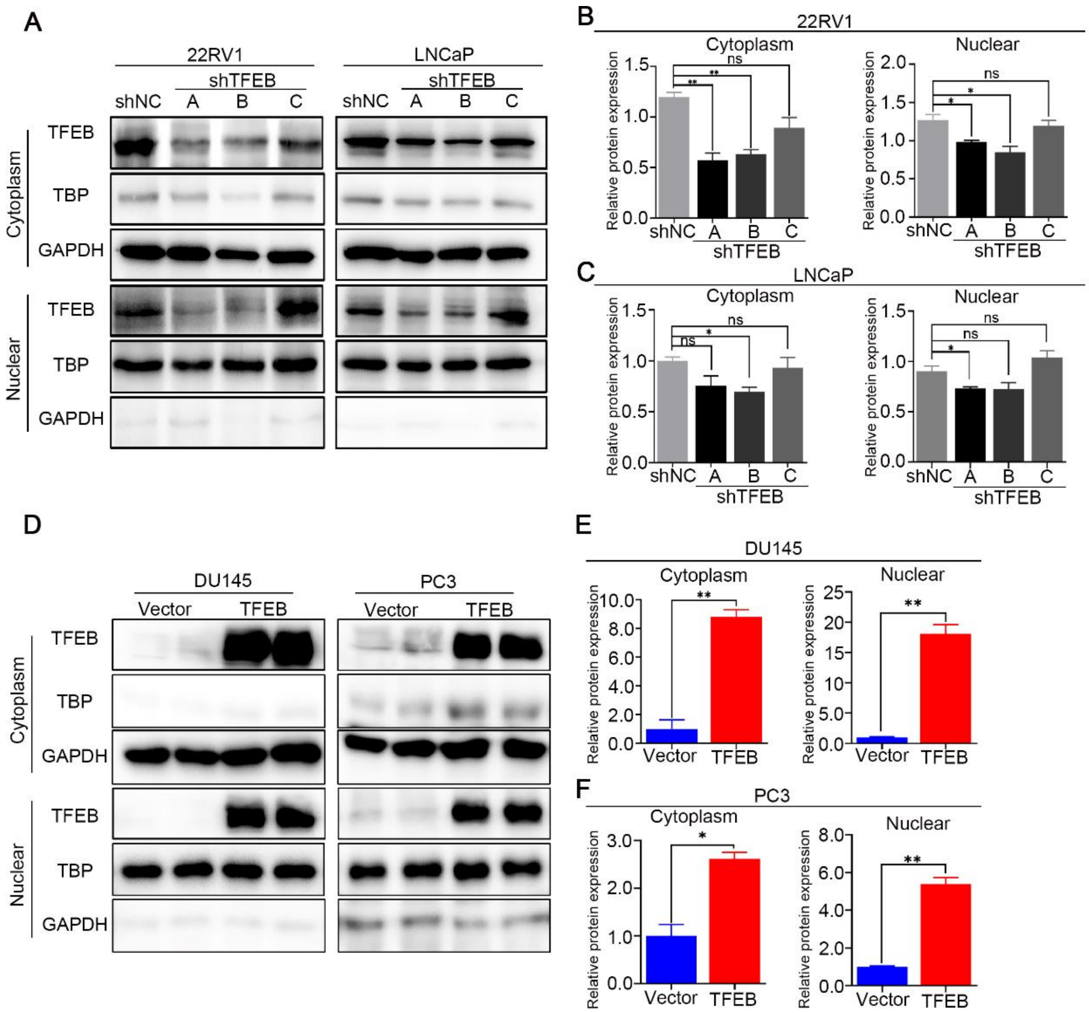

Legends

Supplementary Figure 1. Validation of TFEB transfected PCa cell lines.

(A) The expression of TFEB was knockdown in 22RV1 and LNCaP cell lines. The protein levels of TFEB in cytoplasm and nuclear was performed by western blot respectively.

(B) Protein quantitation of TFEB in 22RV1 cells cytoplasm and nuclear.

(C) Protein quantitation of TFEB in LNCaP cells cytoplasm and nuclear.

(D) The expression of TFEB was overexpressed in DU145 and PC3 cell lines. The protein levels of TFEB in cytoplasm and nuclear was performed by western blot respectively.

(E) Protein quantitation of TFEB in DU145 cells cytoplasm and nuclear.

(F) Protein quantitation of TFEB in PC3 cells cytoplasm and nuclear.

Note: Statistical analysis was from three independent experiments and is presented as mean ± SD. *p < 0.05 and **p<0.01 compared with control group.
