# Supplementary material for: TFEB Promotes Prostate Cancer Progression via Regulating ABCA2-Dependent Lysosomal Biogenesis
Source: Front Oncol. 2021 Mar 1;11:632524. doi: 10.3389/fonc.2021.632524 (PMC7959325; doi:10.3389/fonc.2021.632524)
Supplement: Supplementary file 3 [file DataSheet_3.pdf]

# *Supplementary Figure 2*

**Supplementary Figure 2**

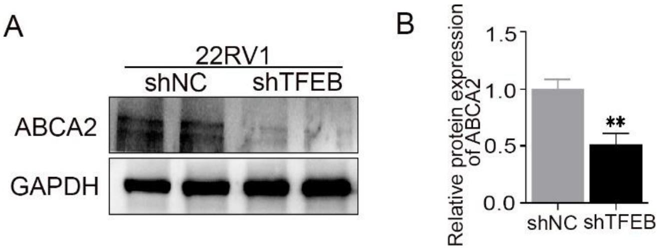


**Legends**

**Supplementary Figure 2. Knockdown TFEB**

**(A)** Western-blot verification of ABCA2 gene in TFEB knockdown cell lines.

**(B)** Quantitative analysis of the western-blot.
